# An automatic densimeter system for the measurement of the alcoholic strength of potable spirits

**DOI:** 10.1155/S1463924681000424

**Published:** 1981

**Authors:** R. G. Lidzey, B. M. Stockton, M. E. B. Brown

**Affiliations:** 1 Laboratory of the Government Chemist Cornwall House Stamford Street London SE1 9NQ UK; 2 Plasma-Therm Ltd 6 Station Road Penge London SE20 7BQ UK


### An automatic densimeter system for the measurement of the alcoholic strength of potable spirits

**R. G. Lidzey, B. M. Stockton, M. E. B. Brown***
*Laboratory of the Government Chemist, Cornwall House, Stamford Street, London SE1 9NQ*

### Introduction

Large numbers of samples of alcoholic liquors are received in the Laboratory of the Government Chemist from HM Customs and Excise every year and the majority require a simple alcoholic strength measurement. Determination of the alcohol strength of spirituous beverages is usually carried out by distillation followed by density measurement of the distillate. For fiscal purposes, traces of volatile fermentation by-products in the distillate are deemed to contribute to the strength and the density values are referred to alcohol/water v density tables issued by HM Customs and Excise [1].

There are four steps in the analytical procedure: sampling a known aliquot at 20°C, its distillation, measurement at 20°C of the volume of distillate obtained and estimation of its density. Automation of the first three steps is difficult, samples vary greatly in viscosity and the more viscous liquors would not pass through the passageways of most dispensing arrangements. Also, by using a constant temperature bath and a bank of four or more stills, one operator can produce distillates ready for density measurement at a rate of about 20 per hour, which would be hard to match with an automatic system.

The normal UK method [2] for the measurement of density is by using the pycnometer. A glass bottle, fitted with a ground glass stopper that is pierced with a central capillary hole, defines the volume. Liquid is ejected through the capillary when the stopper is inserted and the surface of the stopper where the capillary emerges is wiped dry. The bottle and its contents are then weighed to determine the density. This technique requires several weeks training for the operator before consistent results are obtained and can suffer from operator bias. It was therefore considered to be the best part of the procedure to automate.

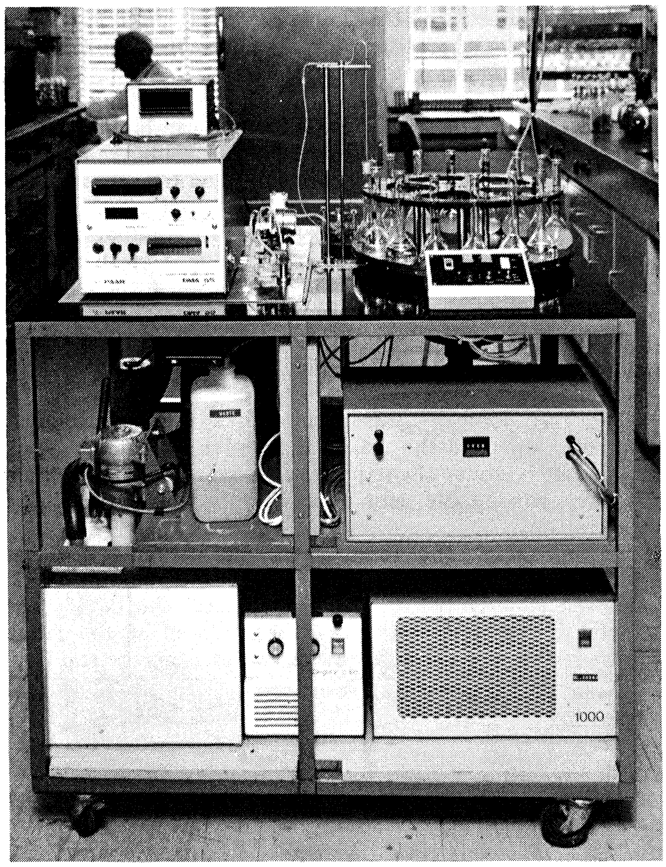

*Figure 1. General view of the automatic assembly.*

### Apparatus

### Instrumentation

A number of instrumental methods [3,4,5] have been developed in recent years which rely on the magnetic suspension of a float for measurement, and are suitable for the range, temperature and sensitivity required for measuring alcohol solutions. An automatic instrument described elsewhere [6] has been constructed in this laboratory. Samples and standards are sequentially introduced by a mechanism into a pre-cooler and then into a thermostatted cell where the deflection of a float supported by a stainless steel wire is related to the solution density. Daily calibration of this instrument with alcohol and water standards is time consuming and advantage was taken of the recent introduction of the Anton Paar DMA-55 [7], which requires only air and water as references to integrate it into an improved version of the existing automatic system. The DMA-55 and an earlier model have already been used in automatic systems for the examination of wines and spirits [8] and also beer [9] in other countries.

The measuring principle for the instrument depends upon the variation of the natural frequency of oscillation of a hollow U tube which is filled with the test liquid. The period of oscillation of the tube is given as:

$$T = 2\pi\sqrt{\frac{\rho . V + M}{c}}$$

where T = the period.
M = the mass of the tube.
c = a constant related to the elasticity of the tube.
V = volume of the tube.
$\rho$ = the density of the liquid.

Either the density or period of oscillation may be read off from a digital display.

For the purposes of calibration, the period T is measured when the tube is filled with distilled water and also with air.

**Present address:*
*Plasma-Therm Ltd, 6 Station Road, Penge, London SE20 7BQ*

The period is read off and two constants A and B are calculated according to the equations:

$$A = \frac{T^2\ (\text{water}) - T^2\ (\text{air})}{\rho\ (\text{water}) - \rho\ (\text{air})}$$

$$B = T^2\ (\text{air}) - A.\rho\ (\text{air})$$

The A and B values are set on the instrument by means of thumb-wheel switches. After this calibration, displayed values of density may be read off with a precision of $\pm$ 1 x $10^{-5}$ g/cm$^3$ when the U tube or cell is filled with a sample liquid for any value between zero and 1.5 x the density of water.

**Automation of the instrument**

The automatic assembly is constructed as a single free-standing unit (see Figure 1) exlcuding the printer. It contains (see Figure 2) a turntable sample-carrier, a sampling system, a precooler to adjust the sample temperature to approximately 20°C, two peristaltic pumps and a manually operated valve for introducing air and acetone for drying the cell. When the valve is open a few millilitres of acetone can be introduced into the air stream by the dispenser which removes aqueous traces in the cell from any previous test and the acetone then evaporates in the air stream.

The turntable holds up to 16 x 100 cm$^3$ volumetric flasks that are sealed with a plasticised PVC cling wrap film which is pierced by a pneumatically operated probe at the sampling station. The flask volume is a convenient size as it can serve to hold distillates prepared from 25, 50 and 100 cm$^3$ of sample, or the original sample if the density of this is required.

The test solution passes through a pump, precooler, debubbler and into the cell in that order so that the liquid in the cell is under slight positive pressure and there is no tendency for bubble generation as this would vitiate the measurement.

To encourage the removal of the previous distillate solution from the line leading to the cell, the pumping action is started slightly before the probe enters the liquid. The bubble of air thus introduced is removed at the pump together with any entrained air displaced from the solution. The debubbler is in the form of an inverted U-tube with an internal diameter of approximately 0.6 mm at the top. A connection leads to a smaller peristaltic pump which removes air collecting here at about 1 cm$^3$ per minute when the main pump is running. Heavy-wall silicone rubber pump tubing (Cole Parmer 6411-43*) is used for both the main pump (Cole Parmer : Masterflex-7014*) and for the smaller pump (Ismatic mini-micro-2 : Frost Instruments†). This combination runs for several months before the tubing needs to be changed.

The precooler consists of a 10-turn helix of thin walled silver tubing, constructional details of which have been published previously [10]. (Briefly, a solid solder coil is electroplated with a layer of silver. The solder is removed by melting to leave the silver coil). The coil is situated in the temperature-controlled effluent water stream of the cell and brings the temperature of that test liquid from ambient to within 0.1°C of the cell temperature.

Under automatic operation the distillate is pumped at 30 cm$^3$/minute through the cell. The period of pumping can be adjusted for optimum wash characteristics, commensurate with available volume and minimal process time, by thumb-wheel switches at a control panel. A delay period, set by thumb-wheel switches, is introduced to allow the liquid to equilibrate at the cell temperature. Suitable periods of pumping and delay times are currently set at 1 and 2.5 minutes respectively. The flow path has a maximum internal diameter of 1.5 mm except at the bubble trap which has a dead volume of about 0.5 ml. Connection between devices is by 1.5 mm OD PTFE tubing.

Control of the automatic process is by microprocessor (see Figure 3) using an Intel 8080 CPU which, besides controlling the switch gear for the pump, probe operation and turntable rotation also transfers the density value from a BCD outlet on the Paar instrument to a nearby printer after the equilibrium delay time. The 600 step machine code program is held in UV erasable memory so that adjustments can easily be made to the sequence if desirable. A flow chart

*Cole Parmer Instrument Co Ltd, PO Box 22, Bishop Stortford, Herts, UK*

*†Frost Instruments Ltd, Fishponds Rd, Woking, Berks, UK*

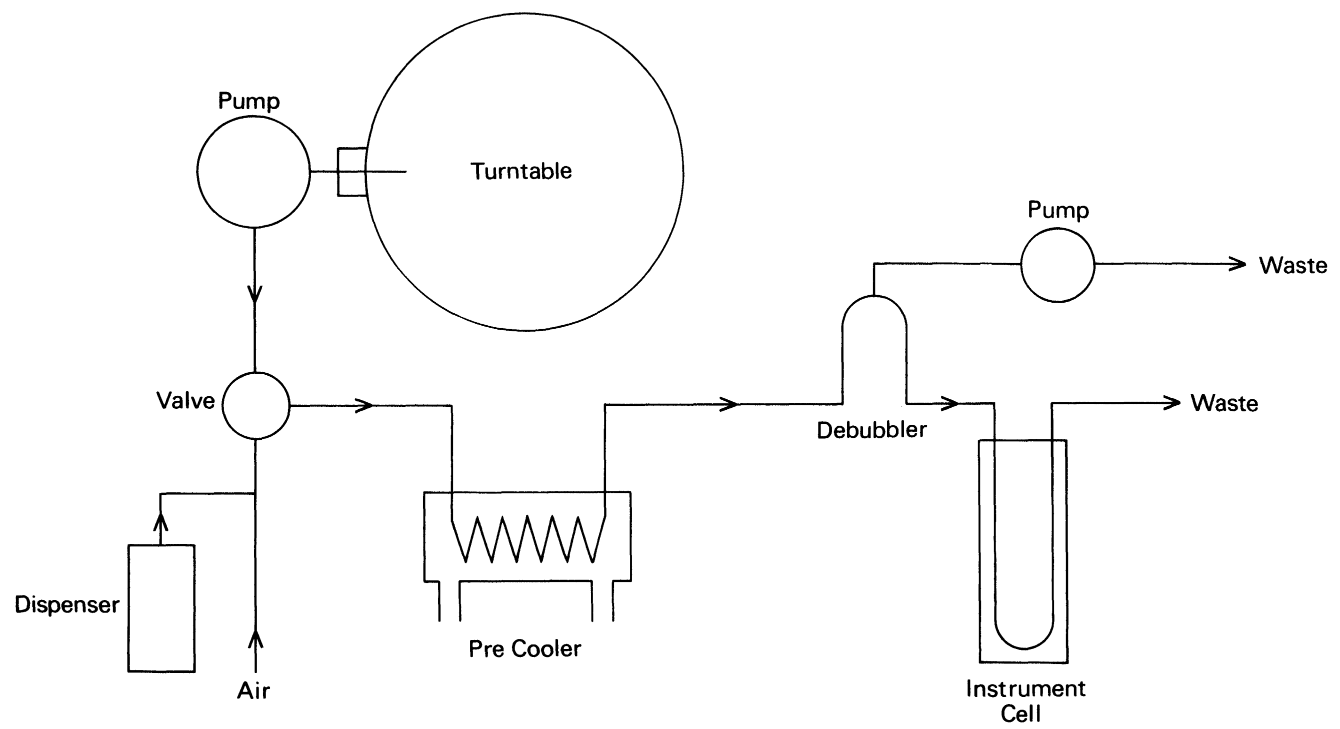

*Figure 2. Flow paths of air and liquid when drying and filling the cell.*

(see Figure 4) indicates the steps followed in the automatic sequence.

A control panel allows manual operation of the pump and sample probe and the turntable can be freely rotated to position and distillate under the probe for special sampling conditions or before the start of the automatic sequence. One position on the turntable is designated the last sample position and the automatic transfer of distillates to the cell stops after the sample in this position is examined. The alignment of sample vessel with the probe and the detection of the last sample is secured by optical detectors beneath the turntable.

To monitor the temperature of the cell, which is particularly important with alcohol solutions, a digital thermometer [11] senses the mean temperature of the inlet and outlet temperature controlling water streams to the cell.

In practice the thermostatic control and densimeter are allowed a period of 1 to 2 hours for temperatures to stabilise before use. Some of the distillates have an oily nature and the cell and lines are filled with a detergent solution when the instrument is not in use. The detergent is washed out and the cell is dried and a value for the air density read off from the display. A further density reading is then taken after the cell is filled with distilled water. It has been assumed, following OIML [12], for the purposes of calibration of the DMA-55, that when measured in air the density of air is zero and the density of water is 0.99715 g/cm$^3$, unless the values given by the instrument differ by more than $\pm 1 \times 10^{-5}$ from these values respectively. No recalculations of the constants A and B for resetting the instrument need to be made. To check on the behaviour of the instrument during the day a flask containing distilled water is added to each turntable of distallates.

### Results and discussion

A number of distillates and also some residues from brandy distillations were examined, first using the density bottle and then by the automatic technique (Table 1). There was

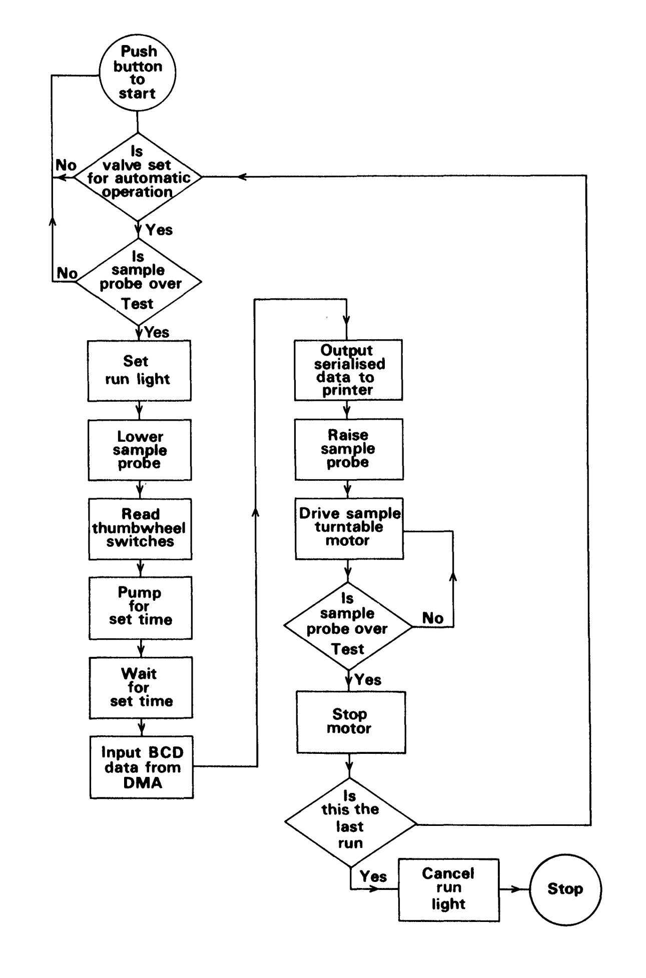

*Figure 4. Flow path for operational sequence.*

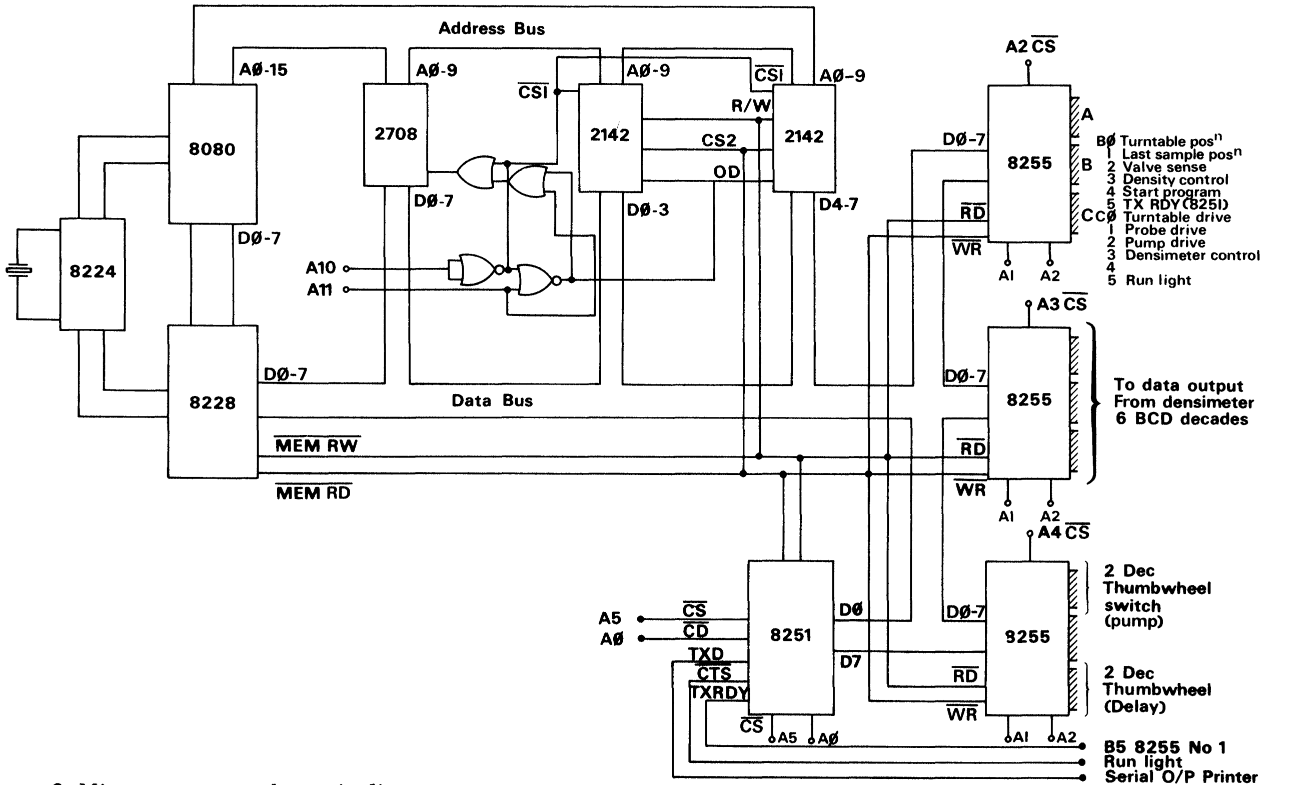

*Figure 3. Microprocessor schematic diagram.*

**Table 1. Results obtained by manual and automated method**

| Operator 1 | Manual | Densimeter | Difference |
|---|---|---|---|
| Sample 1 | 0.96342 | 0.96346 | +4 x $10^{-5}$ |
| 2 | 0.97110 | 0.97111 | +1 |
| 3 | 0.97115 | 0.97113 | −2 |
| 4 | 0.98051 | 0.98050 | −1 |
| 5 | 0.98070 | 0.98069 | −1 |
| 6 | 0.96230 | 0.96235 | +5 |
| 7 | 0.96311 | 0.96312 | +1 |
| 8 | 0.96211 | 0.96211 | 0 |
| 9 | 0.98193 | 0.98192 | −1 |
| 10 | 0.97283 | 0.97281 | −2 |
| 11 | 0.96967 | 0.96969 | +2 |
| 12 | 0.96994 | 0.96993 | −1 |
| Operator 2 | | | |
| Sample 1 | 1.00085 | 1.00085 | 0 |
| 2 | 1.00097 | 1.00094 | −3 |
| 3 | 1.00039 | 1.00037 | −2 |
| 4 | 0.99958 | 0.99954 | −4 |
| 5 | 0.99920 | 0.99916 | −4 |
| 6 | 0.99903 | 0.99901 | −2 |
| 7 | 0.95066 | 0.95067 | +1 |
| 8 | 0.95078 | 0.95080 | +2 |
| 9 | 0.94989 | 0.94996 | +7 |
| 10 | 0.94899 | 0.94902 | +3 |
| 11 | 0.94883 | 0.94892 | +9 |
| 12 | 0.94895 | 0.94898 | +3 |
| Operator 3 | | | |
| Sample 1 | 0.98474 | 0.98475 | +1 |
| 2 | 0.98673 | 0.98676 | +3 |
| 3 | 0.98678 | 0.98679 | +1 |
| 4 | 0.98576 | 0.98577 | +1 |
| 5 | 0.97888 | 0.97898 | +10 |
| 6 | 0.98459 | 0.98467 | +8 |
| 7 | 0.97131 | 0.97130 | −1 |
| 8 | 0.97113 | 0.97118 | +5 |
| 9 | 0.96975 | 0.96971 | −4 |
| 10 | 0.97285 | 0.97293 | +8 |
| 11 | 0.98284 | 0.98287 | +3 |
| 12 | 0.96696 | 0.96697 | −1 |

**Table 2. Analysis of variance**

| Source of variation | Sums of squares | Degrees of freedom | Mean square | F-ratio |
|---|---|---|---|---|
| Between methods | 72.25 | 1 | 72.250 | 3.13 ns |
| Between operators | 46.17 | 2 | 23.085 | 1.75 ns |
| Residual | 434.58 | 33 | 13.170 | |
| Total | 553.00 | 36 | | |

ns = not significant

Differences are multiplied by $10^5$ for the analysis no bias between the methods (Table 2) and a separate set of distillate solutions examined in duplicate by the automatic instrument showed that the precision is also satisfactory (Table 3).

The system has been in operation for about one year in a laboratory engaged in routine analysis of wines and spirits and is regarded as simple to use and reliable. Building the DMA-55 densimeter into an automatic system has improved the consistency of the results. A number of factors may have contributed to this. Automatic filling of the cell now prevents the distillate coming into contact with air and stops the consequential loss of alcohol, which can occur when filling the syringe. Also the inadvertent introduction of air into the cell has been reduced by using a bubble trap. The constant period between filling the cell and printing out the density value also helps consistency. Finally, pumping a comparatively large volume of distallate (30 $cm^3$) through the cell which has a volume of only 0.7 $cm^3$, probably is more reliable than flushing the cell with separate small portions of test liquid.

Eventually it is intended to print out alcohol strength as well as density. This will make it necessary to code each sample position for sample/distillate bulk ratio so that the correct factor can be used for calculating strength from density.

**Table 3. Densimeter readings**

| Sample | Readings 1 | Readings 2 |
|---|---|---|
| 1 | 0.98350 | 0.98349 |
| 2 | 0.98345 | 0.98344 |
| 3 | 0.98385 | 0.98389 |
| 4 | 0.98381 | 0.98382 |
| 5 | 0.98373 | 0.98373 |
| 6 | 0.98370 | 0.98370 |
| 7 | 0.97780 | 0.97780 |
| 8 | 0.97774 | 0.97774 |
| 9 | 0.98155 | 0.98154 |
| 10 | 0.98154 | 0.98157 |
| 11 | 0.98150 | 0.98150 |
| 12 | 0.98556 | 0.98556 |

**Analysis of variance**

| Source of variation | Sums of squares | Degrees of freedom | Mean square |
|---|---|---|---|
| Between samples | 1.298E-04 | 11 | 1.181E-05 |
| Residual | 1.45 E-09 | 12 | 1.208E-10 |

Standard deviation of the automatic method = 1.1 x $10^{-5}$

REFERENCES

[1] "Laboratory alcohol table : density/strength at 20°C for laboratory use" issued under regulation 49 of the Spirits Regulations 1952, as amended by The Alcohol Liquors (Amendment of Units and Methods of Measurement) Regulations 1979.

[2] "British Standard 733 : 1965", The British Standards Institution London, 1965.

[3] Hodgins, M. G. and Beams, J. W., (1971), *Review Sci Inst*, **42**, 10, 1455-7.

[4] Senter, J. P., (1969), *Review Sci Inst*, **40**, 2, 334-8.

[5] Hayner, W. M.,(1977), *Review Sci Inst*, **48**, 1, 39-41.

[6] Bunting, W. and Stockwell, P. B., (1978), *Analyst*, **103**, 72.

[7] Kratky, O., Leopold, H. and Stabinger, H., (1969), *Z angew Physik*, **4**, 273.

[8] Strunk. D. H., Haman, J. W. and Timmel, B. M., (1979), JAOAC, **62**, 3, 653-8.

[9] Benard, M., (1977), *Brewers Digest*, **52**, 9, 64-66.

[10] Lidzey, R. G., Kallar, D. and Porter, D. G., (1977), *Lab Practice*, **26**, 8, 629.

[11] Brown, M. E. B. and Lidzey, R. G., (1981), *Journal of Automatic Chemistry*, **3**, 1, 38.

[12] Valeur conventionelle du Resultat des Pésées dans L'air, Recommandation Internationale No 33, Bureau International de Métrologie Légale Paris, 1973.

